# Comparative Genome Analysis of *Staphylococcus lugdunensis* Shows Clonal Complex-Dependent Diversity of the Putative Virulence Factor, *ess*/Type VII *Locus*

**DOI:** 10.3389/fmicb.2019.02479

**Published:** 2019-10-31

**Authors:** Jérémie Lebeurre, Sandrine Dahyot, Seydina Diene, Amandine Paulay, Marion Aubourg, Xavier Argemi, Jean-Christophe Giard, Isabelle Tournier, Patrice François, Martine Pestel-Caron

**Affiliations:** ^1^UNIROUEN, GRAM EA2656, Normandie Université, Rouen, France; ^2^UNIROUEN, GRAM EA2656, Rouen University Hospital, Normandie Université, Rouen, France; ^3^Genomic Research Laboratory, Service of Infectious Diseases, Geneva University Hospitals, Geneva, Switzerland; ^4^EA4655 U2RM (Équipe Antibio-Résistance), Université de Caen Basse-Normandie, Caen, France; ^5^CHRU de Strasbourg, VBP EA7290, Fédération de Médecine Translationnelle de Strasbourg (FMTS), Institut de Bactériologie, Université de Strasbourg, Strasbourg, France; ^6^UNIROUEN, Inserm U1245, Normandy Centre for Genomic and Personalized Medicine, Rouen University Hospital, Normandie Université, Rouen, France

**Keywords:** *Staphylococcus lugdunensis*, genomic comparative analysis, virulence, *ess*/type VII *locus*, clonal complexes

## Abstract

*Staphylococcus lugdunensis* is a commensal bacterium of human skin that has emerged as a virulent Coagulase-Negative *Staphylococcus* in both community-acquired and healthcare associated infections. Genotyping methods have shown a clonal population structure of this pathogen but failed to identify hypervirulent lineages. Here, complete genomes of three pathogenic and three carriage *S. lugdunensis* strains were obtained by Single-Molecule sequencing (PacBio) and compared to 15 complete genomes available in GenBank database. The aim was to identify (i) genetic determinants specific to pathogenic or carriage strains or specific to clonal complexes (CCs) defined by MultiLocus Sequence Typing, and (ii) antibiotic resistance genes and new putative virulence factors encoded or not by mobile genetic elements (MGE). Comparative genomic analysis did not show a strict correlation between gene content and the ability of the six strains to cause infections in humans and in a *Galleria mellonella* infection model. However, this study identified new MGEs (five prophages, two genomic islands and one plasmid) and genetic variations of some putative virulence-associated *loci*, especially in CC3 strains. For a clonal population, high variability and eight CC-dependent genetic organizations were observed for the *ess locus*, which encodes a putative type VII secretion system (T7SS) homologous to that of *S. aureus*. Further phenotypic and functional studies are needed to characterize this particular CC3 and to evaluate the role of T7SS in the virulence of *S. lugdunensis*.

## Introduction

*Staphylococcus lugdunensis* is a Coagulase-Negative Staphylococcal species (CoNS) that is a part of the normal human skin flora. However, unlike other CoNS, *S. lugdunensis* exhibits a high degree of virulence similar in some respects to *Staphylococcus aureus* (Frank et al., 2008).

*Staphylococcus lugdunensis* is responsible for infections ranging from minor skin and soft tissue infections (Bocher et al., 2009; Arias et al., 2010) to invasive diseases such as infective endocarditis (Non and Santos, 2017), bone and joint infections (Douiri et al., 2016; Lourtet-Hascoët et al., 2016; Argemi et al., 2017a), prosthetic device-infections (Anguera, 2005; Shah et al., 2010) and vascular catheter-related infections (Ebright et al., 2004), both in healthy host and immune-compromised patients. One of the characteristics of *S. lugdunensis* infections is the development of deep-seated abscess lesions that cannot be cleared without surgical intervention associated with effective antibiotic therapy (Arias et al., 2010). Interestingly, few virulence factors have been described to date to explain the high ability of *S. lugdunensis* to cause acute and suppurative infections, and particularly tissue damage and elevated mortality observed for infections such as infective endocarditis (Anguera, 2005; Sabe et al., 2014).

Several molecular typing methods have been described to search for a potential link between within-species genetic variations and pathogenic potential or virulence. Multi-Locus Sequence Typing (MLST) has shown a clonal population structure of this pathogen (Chassain et al., 2012), and has allowed the description of seven Clonal Complexes (CCs)^1^ but failed to identify hypervirulent lineages or clusters specific to carriage strains. Multi-Virulence Locus Sequence Typing (Didi et al., 2014) as well as Multiple Locus Variable Number Tandem Repeat Analysis (MLVA) and Tandem Repeat Sequence Typing (TRST) recently developed, have confirmed this absence of link between clustering and clinical settings (Dahyot et al., 2018).

Whole-genome sequencing (WGS) has become a powerful tool in microbiology allowing the sequencing of hundreds of *S. aureus* genomes but to date only 15 complete genomes of *S. lugdunensis* are available at the National Center for Biotechnology Information (NCBI). The only two studies comparing *S. lugdunensis* whole genomes revealed that the genome (i) is closer to that of *S. aureus* than other CoNS, (ii) harbors mobile genetic elements (MGE) such as prophages and plasmids, but (iii) displays a closed pan-genome and several systems (restriction-modification, toxin/antitoxin and CRISPR/Cas systems) to prevent horizontal gene transfer (Argemi et al., 2017b, 2018).

In this context, we first sequenced the whole genome of three pathogenic and three carriage strains, previously characterized by MLST (Didi et al., 2014) and collected from four geographic origins in France and Sweden. These whole-genome sequences were compared with data from 15 genomes available in the GenBank database to identify genetic variations specific to pathogenic or carriage strains or to CCs, antibiotic resistance genes and new putative virulence factors carried or not by MGE.

Comparative genomic analysis did not show correlation between gene content and the ability of the six strains to cause infections in humans and in a *Galleria mellonella* infection model. It showed CC-dependent genetic variations of some putative virulence-associated *loci* among 21 genomes analyzed. Surprisingly for a highly clonal population, a great diversity was observed for the *ess locus*, which putatively encodes a T7SS homologous to that of *S. aureus* in which this multiprotein complex plays a key role in pathogenesis. Indeed, it promotes chronically long-term persistence infections with abscesses, lesions and lethal outcomes in a mouse model (Burts et al., 2005, 2008).

## Materials and Methods

### *G. mellonella* Infection Experiments

Infection of *G. mellonella* larvae with *S. lugdunensis* was performed as previously described (Michaux et al., 2011). Briefly, using a syringe pump (KD Scientific, Holliston, MA, United States), larvae (about 0.3 g and 3 cm in length) were infected subcutaneously with washed cells of *S. lugdunensis* from an overnight culture in Brain Heart Infusion, with 3(±2) × 10^6^ CFU per larva administered in 10 μl of sterile saline buffer. In each test, 10 insects were infected, and experiments were repeated at least four times. Larval killing was then monitored at 1-, 2- and 3-days post-infection. Sterile saline buffer was also tested under the same conditions as control. Results were analyzed using one-way analysis of variance with a Bonferroni correction with free “R” software. For all comparisons, a *p* value <0.05 was considered significant.

### Pacific Biosciences Sequencing

The complete genome of three pathogenic and three carriage strains of *S. lugdunensis* previously characterized by MLST and Multi-Virulence Locus Sequence Typing (Chassain et al., 2012; Didi et al., 2014) was sequenced using Single Molecule sequencing Pacific Biosciences technology (Gupta, 2008). Strains SL13, SL29 and SL55 were, respectively, isolated from infective endocarditis, vascular prosthesis infection and skin and soft tissue infection. Carriage strains were isolated from groin (SL118 and 122) and toe (SL117) (van der Mee-Marquet et al., 2003; Bieber and Kahlmeter, 2010). Four strains belonged to CC1 (SL13, SL29, SL117, SL122) and 2 to CC6, as defined by MLST (Chassain et al., 2012; Table 1).

**TABLE 1:** Whole genome sequence content of the 6 *S. lugdunensis* strains sequenced with Pacific Biosciences technology and the 15 *S. lugdunensis* genomes available on NCBI.

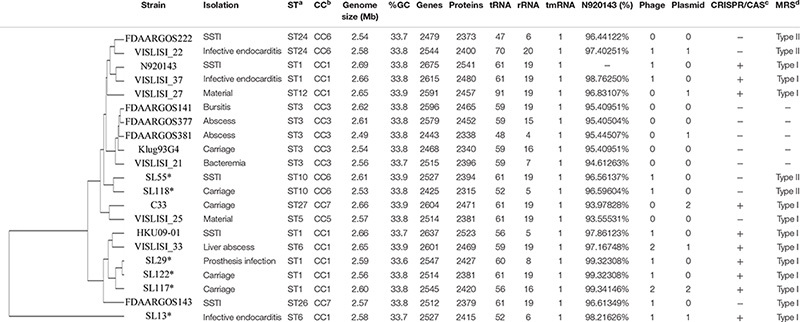

*^*a*^Sequence type. ^*b*^Clonal complex. ^*c*^Clustered, regularly interspaced, short, palindromic repeat-associated (*cas*) genes. ^*d*^Modification-restriction system. ^∗^Genomes sequenced in this study. SSTI, skin and soft tissue infection.*

High molecular weight genomic DNA was purified from 20-ml exponential-phase cultures by using genomic-tip-500g kit (Qiagen) following manufacturer’s instructions. DNA was precipitated in isopropanol, extensively washed with cold 75% ethanol and in 1 × Tris-EDTA buffer. Purity and quantity were assessed by using NanoDrop and Qubit assays, respectively, before size evaluation with a Tape Station (Agilent). DNA was sequenced on a Pacific Biosciences RS system at the Lausanne Genomic Technologies Facility, Center for Integrative Genomics. Using P6-C4 chemistry, libraries of approximately 8–20 kb were generated. Analyses of reads were carried out by using SMRT pipe version 2.3.0 software (Pacific Bioscience). The quality value (QV) threshold for motif calling was set at 30.

### Computational Analysis

In addition to the 6 genomes sequenced by PacBio, 15 complete genomes published or available were retrieved from GenBank for comparative analyses (Supplementary Table S1). All complete genomes were annotated using the software Prokka v1.13 (Seemann, 2014). Genomes were compared using orthologsorter^2^ to generate ortholog clustering sequences among the overall 21 strains and to define the pan-genome, the core-, and the accessory-genome of *S. lugdunensis*. Genomes were also compared according to the clinical context (infection vs. carriage) and the CC of strains. A similarity matrix of complete genomes and a phylogenetic tree were created based on the neighbor-joining method with the software BioNumerics v7.6. Sequence Types (STs) and CCs defined by MLST were identified by extracting sequences of genes *aroE, dat, ddl, gmk, ldh, recA* and *yqiL* (Chassain et al., 2012). STs and CCs were obtained by submission of gene sequences into the international MLST database^3^. The presence of genes encoding restriction enzymes was predicted by comparison and Blastn with datasets of the Restriction Enzyme Database (Roberts et al., 2013).

Integrated prophage regions were identified and annotated by using the online PHAge Search Tool Enhanced Release (PHASTER) (Arndt et al., 2016)^4^ with default parameters for the six genomes sequenced by PacBio, the complete genome sequences of the 5 FDAARGOS strains and the Klug93-G4 strain, available on NCBI, and for which no phage has been described to date. PHASTER provided the region length and position of the prophages on the chromosome, their GC content and the most common related phages. Known prophage sequences ΦSL1 (Heilbronner et al., 2011), ΦSL2, ΦSL3, ΦSL4 and ΦSL5 (Argemi et al., 2017b) were compared with prophage regions identified and Mauve alignments were used to confirm PHASTER results.

Plasmid identification was done by using Prokka v1.13 for annotation of extra-chromosomic contigs obtained by PacBio sequencing and was compared to plasmid sequences retrieved from the NCBI database (pVISLISI_1, pVISLISI_2, pVISLISI_3, pVISLISI_4 pVISLISI_5 (Argemi et al., 2017b) and to unnamed plasmid from the FDAARGOS381 strain). Nucleotide sequence similarities were investigated by Blastn to identify the closest related plasmid.

Virulence factors were identified using the Virulence Factors database (VFDB)^5^ (Chen et al., 2016) and tBlastn searches were performed to compare the presence or absence of known virulence genes of staphylococci. Comparisons of *agr* and *cap* hypervariable regions were performed by extracting and aligning *agr* and *cap loci* with MUSCLE. Allelic *agr* type-I (*agrSl-*type I, accession number: AF173933*)* and type-II *(agrSl-*type II, accession number: AF346728) described by Dufour et al. (2002) were used as reference. The hypervariable regions (*capH to capK*) from *S. aureus cap8* and *cap5 loci* were downloaded from the NCBI database. Phylogenetic trees were constructed using the maximum likelihood method implemented in the PhyML program v3.0.

Resistance genes were identified by using ResFinder v3.1^6^ (Zankari et al., 2012). A cutoff of 80% minimum length and 80% identity were used to search for resistance genes in the 21 genomes and plasmids of *S. lugdunensis*.

Comparisons of individual sequences of the *ess locus* were performed on the 21 genomes of *S. lugdunensis* by using Blastn and Blastp against the NCBI database, and were facilitated by using the Artemis Comparison Tool (Carver et al., 2005). T7SS genes and proteins were identified from available genomes of HO 5096 0412 (Accession number HE861097) (Holden et al., 2013) and RJ-ST398 reference strains (Accession number AM990992) (Wang et al., 2016). Pfam database was also used to identify protein domains.

## Results

### *G. mellonella* Infection Experiments

In order to evaluate and compare the virulence of 6 *S. lugdunensis* strains (SL13, SL29, SL55, SL117, SL118 and SL122) under the same conditions *in vivo*, a *G. mellonella* model of infection was used.

**FIGURE 1:**
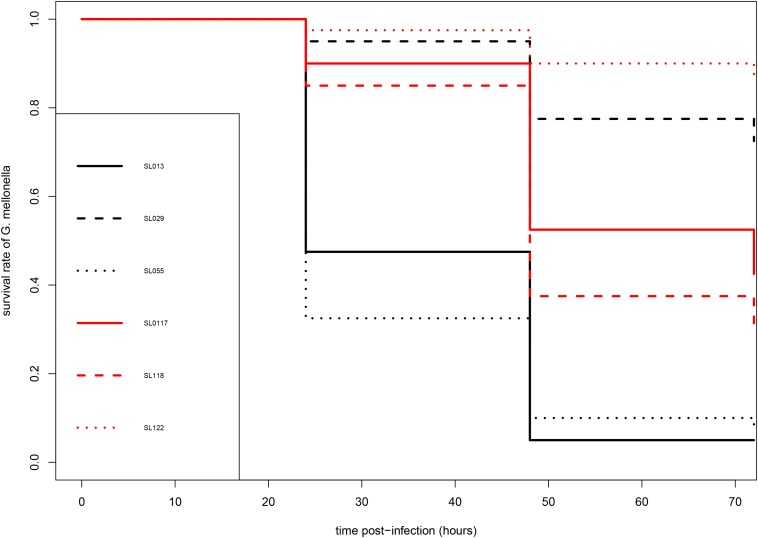
Kaplan-Meier survival curves of infected larvae of *G. mellonella* after infection by *S. lugdunensis* strains. Pathogenic (SL13, SL29, SL55) and carriage (SL117, SL118, SL122) are shown in black and red, respectively.

As shown in Figure 1, carriage isolates (SL117, SL118, and SL122) as well as the pathogenic isolate SL29 (from vascular prosthesis infection) revealed moderate virulence in this model of infection. After 72 h, 31–87% of the larvae infected by these four isolates were still alive. However, they could not be clustered because statistical analysis displayed significant differences between them (*p* values <0.05) (Table 2). In contrast, isolates SL13 (from infective endocarditis) and SL55 (from a skin and soft tissue infection) were significantly more virulent than the others since only 4 and 8% of the infected caterpillars survived 3-days post infection, respectively (Figure 1 and Table 2). No mortality was observed for the larvae into which sterile saline buffer was injected (data not shown). Of note, no differences were observed between strains in terms of colony morphology, hemolysis (β-hemolysis on blood agar) and growth characteristics under standard laboratory conditions.

**TABLE 2:** Pair test statistical comparison (*p* values) of the virulence of 6 strains of *S. lugdunensis* in a *Galleria mellonella* infection model.

	**SL13**	**SL29**	**SL55**	**SL117**	**SL118**
SL29	2.14e-10	NA	NA	NA	NA
SL55	1.00	1.71e-10	NA	NA	NA
SL117	5.72e-06	0.11	4.33e-06	NA	NA
SL118	0.0006	0.002	0.0003	1.00	NA
SL122	2.12e-13	1.00	1.52e-13	0.0004	2.68e-06

### Pacific Biosciences Genome Sequences and Functional Genome Annotation

PacBio sequencing of 6 *S. lugdunensis* strains resulted in full genome recovery on a single contig with sequence length ranging from 2.53 (SL118) to 2.61 Mb (SL55). Between 67457 (SL55) and 79450 (SL118) post-filter polymerase reads were generated with an average read length ranging from 13.7 (SL55) to 14.8 kb (SL13). The N50 read length was comprised between 19.2 and 22.7 kb, and the average coverage between 272× and 367×. In addition to the contigs corresponding to genomes, 0 (SL29 and SL118) to 3 (SL117) extra-chromosomic contigs were generated.

The complete genome sequences of 15 different *S. lugdunensis* strains available in the NCBI database (Supplementary Table S1) were compared to the six genomes sequenced by PacBio. The size of these 21 complete genomes ranged between 2.53 (SL118) and 2.69 Mb (N920143). All strains presented similar %GC-content (33.6–33.9%). The number of genes automatically predicted by Prokka software ranged from 2443 (FDAARGOS381) to 2675 (N920143) genes encoding between 2338 and 2541 proteins. Genomic data for the 21 strains are shown in Table 1.

### Pan-, Core- and Accessory-Genome Analysis

Pan-genome analysis led to identification of the total gene repertoire of the species and sets of core and accessory genes available in each *S. lugdunensis* genome. The pan-genome of the 21 *S. lugdunensis* strains was found to be composed of a total repertoire of 3077 genes. Of these, 2185 genes (71.01%) were part of the core-genome, and 892 genes (28.99%) were part of the accessory genome. Based on this analysis, no gene retrieved from the accessory genome belonged only to pathogenic or only to carriage strains. In the same way, no gene was specifically associated with strains isolated from a deep infection such as infectious endocarditis, deep abscess, or device-associated infection.

However, CC-dependent genetic variations were observed. Accessory-genome analysis of CC1 strains revealed that 18 genes were unique to these nine strains. Among them, nine genes were clustered, regularly interspaced, short, palindromic repeat (CRISPR) and *loci*-associated corresponding to type III-A proteins: Cas1, Cas2, Cas10, Csm2, Csm3, Csm4, Csm5, Csm6, and Cas6. The *cas locus* from CC1 strains showed a sequence homology of 76% with that of *S. epidermidis* RP62a strain. In all CC1 strains, except strains SL29 and SL122, a potential frameshift is located in the gene encoding the Csm6 protein and probably results in a non-functional CRISPR/Cas system. The remaining nine genes unique to CC1 strains were carried by a transposon and encode arsenical resistance proteins.

Eight genes were unique to CC6 strains. Six of them encode hypothetical proteins but two encode a type II restriction-modification system (RMS). Indeed, we identified a C-5 cytosine-specific DNA methylase and a Type-2 restriction enzyme *Sau*3AI sharing 82% identity with RMS type II from *S. haemolyticus* JCSJ1435 strain. Only 2 genes were found specific to CC7 strains: one encoding a DUF600 protein and one encoding a hypothetical protein. No gene was identified as specific to CC3 strains.

### Mobile Genetic Elements

Five new intact prophage regions named ΦSL6 to ΦSL10 were identified by PHASTER analysis for 12 of the 21 strains (Table 3). Prophage regions ΦSL6, ΦSL7 and ΦSL8 were identified in CC1 strains (SL13, SL29, SL117, and SL122) while prophage regions ΦSL9 and ΦSL10 were found in CC6 (SL55 and SL118) and CC7 (FDAARGOS143) strains, respectively. The total length of these prophages ranged from 46.6 kb (ΦSL9) to 49.9 kb (ΦSL7); they encoded 26–69 proteins. Among these proteins, no virulence factors or antibiotic resistance-associated proteins were identified. However, we identified a putative bifunctional autolysin, a cell wall hydrolase lytN precursor, a collagen triple-helix protein and a LexA repressor which might be involved in the remodeling of the bacterial cell wall.

**TABLE 3:** Prophage regions and plasmids identified among 21 genomes of *S. lugdunensis*.

	**Prophages**					**Plasmids**		
		
	**ΦSL6**	**ΦSL7**	**ΦSL8**	**ΦSL9**	**ΦSL10**	**pSL1**	**pSL2**	**pSL3**
Host strain	SL13	SL29^∗^, SL117, SL122	SL117	SL55, SL118	F143	SL13	SL117	SL117
Length (kb)	45.9	49.9	49.1	46.6	57	2.7	3.9	1.9
GC%	34.94	35.33	35.3	34.45	35.24	30.59	28.99	31.3
Coding sequences	51	26	62	69	61	4	5	4
Virulence factor gene	0	0	0	0	0	0	0	0
Resistance gene	0	0	0	0	0	*tet(k)/tet(L)*	*cadC*	0
Related phage	187	Stb12	CNPx	CNPx	CNPH82	–	–	–
Original host	*S. aureus*	*S. hominis*	*S. epidermidis*	*S. epidermidis*	*S. epidermidis*	–	–	–
Related plasmid	–	–	–	–	–	pSSTET1	pLUG10	pVISLISI_2
Identities with the related plasmid (%)	–	–	–	–	–	99%	98%	100%
Coverage (%)	–	–	–	–	–	100%	48%	100%

***^∗^**duplication.*

Functional annotation by using Prokka on additional contigs led to identification of three plasmids in two of the six newly sequenced genomes: pSL1 in strain SL13, and pSL2 and pSL3 in strain SL117 (Table 3). The total length of the plasmids identified ranged from 1.9 (pSL3) to 3.9 kb (pSL1); they encode between 4 and 5 proteins. Plasmids pSL2 and pSL3 respectively correspond to cadmium resistance-associated plasmid pLUG10 (Poitevin-Later et al., 1992) and plasmid pVISLISI_2 (Argemi et al., 2017b) previously described. Of note, no virulence factor was carried on these plasmids but tetracycline and cadmium resistance genes were found.

In addition, we identified a region of 13 kb length restricted to CC1 strains SL13 and VISLISI_33. Around 5 kb of this region shared a sequence homology of 94% with the pathogenicity islands SaPITokyo11212 and SaPIivm60 of *S. aureus* strains Tokyo11212 and IVM60, respectively. This region encodes an integrase, an excisionase, a primase and a terminase. Other interesting genes were carried by this putative pathogenicity island as those encoding Ear penicillin binding protein, FhuD ferrichrome ABC transporter and a protein homologous to part of the *S. aureus* Pathogenicity Island (SAPI) domain.

### Resistome

Several antibiotic resistance-associated genes were identified by using the web tool ResFinder v3.0 (Table 4). A gene homologous (83% identity) to the *fosB* gene of *Bacillus cereus* AS4-12 was identified in the chromosome of all *S. lugdunensis* strains. A penicillin resistance-associated gene, *blaZ* was also identified. This gene was carried by a transposon and found in 7 of the 21 *S. lugdunensis* strains. The *blaZ* gene showed a homology ranging from 92 to 100% with the *blaZ* gene carried by the plasmid pSAP106A of *S. epidermidis*. Otherwise, annotation of the extra-chromosomal contig of SL13 strain led to identification of a *tetK* tetracycline resistance gene which shared 99% identity with *tetK* gene from the plasmid pT181 of *S. aureus.* Of note, no gene of methicillin resistance, within a composite staphylococcal chromosome cassette *mec* element, was found.

**TABLE 4:** Antibiotic resistance-associated genes identified among 21 genomes of *S. lugdunensis* and their genomic location.

**Antibiotic**	**Gene**	**Strains**																				**Genomic location**
			
		**SL13**	**SL29**	**SL55**	**SL117**	**SL118**	**SL122**	**HKU09-01**	**N920143**	**V21**	**V22**	**V25**	**V27**	**V33**	**V37**	**C33**	**F141**	**F143**	**F222**	**F377**	**F381**	
Fosfomycin	*fosB*	+	+	+	+	+	+	+	+	+	+	+	+	+	+	+	+	+	+	+	+	Chromosome
Penicillin	*blaZ*	+	−	−	−	−	−	+	−	+		−	+	+	−	−	+	−	−	+	−	Transposon (chromosome)
Fusidic acid	*fusD*	−	−	−	−	−	−	−	−	−		−	+	−	−	−	−	−	−	−	−	Genomic island (chromosome)
Tetracyclin	*tetK*	+	−	−	−	−	−	−	−	−	−	−	−	−	−	−	−	−	−	−	−	Plasmid

Finally, we also identified a fusidic acid resistance gene located in a genomic island of 16 kb, only in strain VISLISI_27. This gene had 91% homology with the *fusB* gene carried by a genomic island (SaRIfusB) of the *S. aureus* CS6-EEFIC strain. In addition, in this genomic island, we identified a gene encoding a putative virulence factor, *vapE* (for virulence-associated protein E). *vapE* gene was also found in a genomic island of 40 kb in the genome of strain HKU09-01 with an identity of 85%. However, Blastn comparison of these two genomic islands showed 80% identity for only 3 kb, suggesting that strains VISLISI_27 and HKU09-01 acquired these regions independently and from different sources.

### Virulome

Analysis of virulence gene composition based on the Virulence Factor Database (VFDB) showed a number of putative virulence factors that were detected in most of the *S. lugdunensis* isolates. To improve the identification of notable features, tblastn searches of well-characterized staphylococcal virulence factors and transcriptional regulators were performed (Table 5). No gene duplication was detected, except in *isd locus* for one strain (HKU09-01) and in *sst locus* for 20 of 21 strains.

**TABLE 5:** Comparison of virulence factors among 21 *S. lugdunensis* strains according to clonal complexes (CCs).

			**CC1**									**CC3**					**CC6**				**CC7**		**CC5**
							
			**ST1**						**ST6**		**ST12**	**ST3**					**ST10**		**ST24**		**ST26**	**ST27**	**ST5**
											
**Function**	**Gene**	**IDs in N920143**	**SL29**	**SL117**	**SL122**	**V37**	**N920**	**HKU**	**SL13**	**V33**	**V27**	**V21**	**FD141**	**FD381**	**FD377**	**Klug93**	**SL55**	**SL118**	**V22**	**FD222**	**FD143**	**C33**	**V25**
Toxins	*hlb*	SLUG_RS00165	+	+	+	+	+	+	+	+	+	+	+	+	+	+	+	+	*FS*	+	+	0	+
	*hlIII*	SLUG_RS04385	+	+	+	+	+	+	+	+	+	+	+	+	+	+	+	+	+	+	+	+	+
	*Streptolysin S-like toxin*	SLUG_RS12635 to SLUG_RS12360	FS (2 genes)	FS (2 genes)	FS (2 genes)	FS (2 genes)	FS (2 genes)	FS (1 gene)	FS (1 gene)	FS (1 gene)	FS (1 gene)	+	+	+	+	+	FS (1 gene)	FS (1 gene)	+	+	+	FS (2 genes)	+
Enzyme	*enolase*	SLUG_RS10200	+	+	+	+	+	+	+	+	+	+	+	+	+	+	+	+	+	+	+	+	+
	*nuc*	SLUG_RS07915	+	+	+	+	+	+	+	+	+	+	+	+	+	+	+	+	+	+	+	+	+
Phenol-soluble modulins	*slushA*	SLUG_RS02135	+	+	+	+	+	+	+	+	+	+	+	+	+	+	+	+	+	+	+	+	+
	*slushB*	SLUG_RS02130	+	+	+	+	+	+	+	+	+	+	+	+	+	+	+	+	+	+	+	+	+
	*slushC*	SLUG_RS02125	+	+	+	+	+	0	+	+	+	+	+	+	+	+	+	+	+	+	+	+	+
	*psm*ε	SLUG_RS12630	+	+	+	+	+	+	+	+	+	+	+	+	+	+	+	+	+	+	+	+	+
HTH transcription factors	*sarA*	SLUG_RS11005	+	+	+	+	+	+	+	+	+	+	+	+	+	+	+	+	+	+	+	+	+
	*sarR*	SLUG_RS03570	+	+	+	+	+	+	+	+	+	+	+	+	+	+	+	+	+	+	+	+	+
	*sarV*	SLUG_RS03670	+	+	+	+	+	+	+	+	+	+	+	+	+	+	+	+	+	+	+	+	+
	*sarX*	SLUG_RS10740	+	+	+	+	+	+	+	+	+	+	+	+	+	+	+	+	+	+	+	+	+
	*sarZ*	SLUG_RS02940	+	+	+	+	+	+	+	+	+	+	+	+	+	+	+	+	+	+	+	+	+
	*mgrA*	SLUG_RS10655	+	+	+	+	+	+	+	+	+	+	+	+	+	+	+	+	+	+	+	+	+
	*rot*	SLUG_RS05910	+	+	+	+	+	+	+	+	+	+	+	+	+	+	+	+	+	+	+	+	+
	*agr locus*	SLUG_RS05025 to SLUG_RS05040	*Type I*	*Type I*	*Type I*	*Type I*	*Type I*	*Type I*	*Type I*	*Type I*	*Type I*	*Type II*	*Type II*	*Type II*	*Type II*	*Type II*	*type I*	*Type I*	*Type I*	*Type I*	*Type II*	*Type II*	*Type I*
	*saeS*	SLUG_RS00580	+	+	+	+	+	+	+	+	+	+	+	+	+	+	+	+	+	+	+	+	+
	*saeR*	SLUG_RS00585	+	+	+	+	+	+	+	+	+	+	+	+	+	+	+	+	+	+	+	+	+
Proteases	*clpB*	SLUG_RS09430	+	+	+	*FS*	+	+	+	+	+	+	+	+	+	+	+	*FS*	+	+	+	+	+
	*clpC*	SLUG_RS11470	+	+	+	+	+	+	+	+	+	+	+	+	+	+	+	+	+	+	+	+	+
	*clpP*	SLUG_RS10240	+	+	+	+	+	+	+	+	+	+	+	+	+	+	+	+	+	+	+	+	+
	*clpL*	SLUG_RS03575	+	+	+	+	+	+	+	+	+	+	+	+	+	+	+	*S*	+	+	+	+	+
	*clpX*	SLUG_RS06345	+	+	+	+	+	+	+	+	+	+	+	+	+	+	+	+	+	+	+	+	+
Iron metabolism	*sir locus*	SLUG_RS00505 to SLUG_RS00515	+	+	+	+	+	+	+	+	+	+	+	+	+	+	+	+	+	+	+	+	+
	*hts locus*	SLUG_RS04350 to SLUG_RS04360	+	+	+	+	+	+	+	+	+	+	+	+	+	+	+	+	+	+	+	+	+
	*sst locus*	SLUG_RS10415 to SLUG_RS10445	+^∗^	+^∗^	+^∗^	+^∗^	+^∗^	+^∗^	+^∗^	+^∗^	+	+^∗^	+^∗^	+^∗^	+^∗^	+^∗^	+^∗^	+^∗^	+^∗^	+^∗^	+^∗^	+^∗^	+^∗^
	*sfaA*	NA	0	0	0	0	0	0	0	0	0	0	0	0	0	0	0	0	0	0	0	0	0
	*sfaB*	SLUG_RS04340	+	+	+	+	+	+	+	+	+	+	+	+	+	+	+	+	+	+	+	+	+
	*sfaC*	SLUG_RS04345	+	+	+	+	+	+	+	+	+	+	+	+	+	+	+	+	+	+	+	+	+
	*sfaD*	NA	0	0	0	0	0	0	0	0	0	0	0	0	0	0	0	0	0	0	0	0	0
	*isd locus*	SLUG_RS00440 to SLUG_RS00500	+	+	+	+	+	+^∗^	+	+	+	+	+	+	+	+	+	+	+	+	+	+	+
	*srtB*	SLUG_RS00490	+	+	+	+	+	+^∗^	+	+	+	+	+	+	+	+	+	+	+	+	+	+	+
	*hrtA*	SLUG_RS03295	+	+	+	+	+	+	+	+	+	+	+	+	+	+	+	+	+	+	+	+	+
	*hrtB*	SLUG_RS03290	+	+	+	+	+	+	+	+	+	+	+	+	+	+	+	+	+	+	+	+	+
	*hssS*	SLUG_RS03285	+	+	+	+	+	+	+	+	+	+	+	+	+	+	+	+	+	+	+	+	+
	*hssR*	SLUG_RS03280	+	+	+	+	+	+	+	+	+	+	+	+	+	+	+	+	+	+	+	+	+
Host immune evasion	*cap PS*	SLUG_RS01555 to SLUG_RS01630	*cap5*	*cap5*	*cap5*	*cap5*	*cap5*	*cap5*	*cap5*	*cap5*	*cap5*	*cap8*	*cap8*	*cap8*	*cap8*	*0*	*cap5*	*cap5*	*cap5*	*cap5*	*cap5*	*cap5*	*cap8*
	*cap PG*	SLUG_RS02240 to SLUG_RS02230	+	+	+	+	+	+	+	+	+	+	+	+	+	+	+	+	+	+	+	+	+
	*oatA*	SLUG_RS02050	+	+	+	+	+	+	+	+	+	+	+	+	+	+	+	+	+	+	+	+	+
	*dlt locus*	SLUG_RS09690 to SLUG_RS09705	+	+	+	+	+	+	+	+	+	+	+	+	+	+	+	+	+	+	+	+	+
	*mprF*	SLUG_RS07750	+	+	+	+	+	+	+	+	+	+	+	+	+	+	+	+	+	+	+	+	+
Adherence	*ica locus*	SLUG_RS00115 to SLUG_RS00130	+	+	+	+	+	+	+	+	+	+	+	+	+	+	*FS icaA*	*FS icaA*	*FS icaA*	*FS icaA*	+	+	+
	*atlL*	SLUG_RS09125	+	+	+	+	+	+	+	+	+	+	+	+	+	+	+	+	+	+	+	+	+
	*ebpS*	SLUG_RS07180	+	+	+	+	+	+	+	+	+	+	+	+	+	+	+	+	+	+	+	+	+
	*fbpA*	SLUG_RS08490	+	+	+	+	+	+	+	+	+	+	+	+	+	+	+	+	+	+	+	+	+
	*srtA*	SLUG_RS02280	+	+	+	+	+	+	+	+	+	+	+	+	+	+	+	+	+	+	+	+	+
	*slsA*	SLUG_RS01690	+	+	+	+	+	+	+	+	+	0	0	0	0	0	+	+	+	S	+	+	0
	*slsB*	SLUG_RS02320	+	+	+	+	+	+	+	+	+	+	+	+	+	+	+	+	+	+	+	+	+
	*slsC*	SLUG_RS01870	+	+	+	+	+	+	+	+	+	+	+	+	+	+	+	+	+	+	+	+	+
	*slsD*	SLUG_RS01695	S	S	S	S	S	S	S	+	S	+	+	+	+	+	S	S	S	S	+	S	S
	*slsE*	SLUG_RS12395	FS	FS	FS	FS	FS	+	FS	FS	+	+	+	+	+	+	+	+	+	+	+	FS	+
	*slsF*	SLUG_RS01465	+	+	+	+	+	+	+	+	+	+	+	+	+	+	+	+	+	+	+	+	+
	*slsG*	SLUG_RS11265	+	+	+	+	+	+	+	+	+	FS	+	+	+	+	+	+	+	+	+	+	FS
MSCRAMMs	*fbl*	SLUG_RS08200	+	+	+	+	+	+	S	S	S	+	+	+	+	+	+	+	+	+	+	+	+
	*vwbl*	SLUG_RS11765	+	+	+	FS	+	+	+	+	+	S	S	S	S	S	+	+	+	+	+	S	S
Lugdunin	*lugR*	SLUG_RS03935	+	+	+	+	+	FS	+	+	+	+	+	+	+	+	+	+	+	+	+	0	0
	*lugA*	SLUG_RS03940	+	+	+	+	+	+	+	+	FS	+	+	FS	+	+	+	+	+	+	+	0	0
	*lugB*	SLUG_RS03950	+	+	+	+	FS	FS	FS	FS	FS	+	+	FS	+	+	FS	FS	+	+	+	0	0
	*lugC*	SLUG_RS03955	+	+	+	+	+	+	FS	FS	+	+	+	FS	+	+	+	+	+	+	+	0	0
	*lugD*	SLUG_RS03965	+	+	+	+	+	+	+	+	+	+	+	+	+	+	+	+	+	+	+	0	0

*The presence of a putative virulence gene is indicated by “+” while absence is indicated by “0.” PS, polysaccharidic; PG, polyglutamic; FS, frameshift; S, non-sens mutation. ^∗^Duplication; NA, not available. Systematic IDs of the genes are those of the *S. lugdunensis* N920143 strain.*

The accessory gene regulator (*agr*) *locus* (*agrA*, *agrB*, *agrC* and *agrD*) was found in all *S. lugdunensis* strains. Phylogenetic analysis based on alignment of the hypervariable *agr* region (*agrB* to *agrC*) led to identification of 2 genetic groups, *agrSl-*types I and II (Supplementary Figure S1A). Of note, all CC3 isolates were clustered with allele *agrSl-*type II whereas all other strains were closer to allele *agrSl-*type I.

In a region of 16 kb in the genome of the 21 *S. lugdunensis* strains, 16 genes homologous to those encoding polysaccharidic capsule type 5 or 8 in *S. aureus* were detected (Supplementary Figure S1B). In *S. lugdunensis*, only the central region composed of *capH, capI, capJ* and *capK* genes, was found different between strains; the 12 other genes of the *cap locus* were conserved. Based on phylogenetic analysis, the cap *locus* of CC3 and VISLISI_25 strains (CC5) was closer to the *cap8 locus* of *S. aureus* while other strains were related to the *cap5 locus*.

In the genome of *S. lugdunensis*, up to seven *sls* genes (*slsA* to *slsG*) were identified. Comparative analysis of the predicted *S. lugdunensis* surface (Sls) proteins revealed important diversity between the 21 *S. lugdunensis* strains. The size of SlsA, varying from 1634 to 5228 amino acids, correlated with the number of IgG-like domain ranging from 8 to 45. Sequence comparison of SlsA proteins using Blastp showed 51–52% identity with the Bhp protein of *S. epidermidis* RP62a and 63–64% with a cell wall associated biofilm Bhp/SesD protein from *Staphylococcus caprae* JMUB590. Pfam analysis of these two staphylococcal proteins also showed the presence of a similar IgG-like domain, with 18 (Bhp) and 50 (Bhp/SesD) repeats. Interestingly, *slsA* gene was absent from the genomes of all CC3 isolates and from FDAARGOS377 isolates (CC5), and a nonsense mutation was detected in the *slsA* gene of the isolate FDAARGOS222 (CC6). Furthermore, in the *slsD* gene, a nonsense mutation located upstream of the region coding the LPXTG motif was found in 14 of the 21 *S. lugdunensis* strains but was not present in CC3 strains or the VISLISI_33 strain (CC1). Sequence comparison of SlsD proteins using Blastp showed an identity of 40 and 32% with SdrF and SdrG adhesins from *S. epidermidis*, respectively. The size of the SlsE predicted protein sequences varied from 2818 to 4166 amino acids among the 21 *S. lugdunensis* strains. Interestingly, a frameshift was located in the *slsE* gene, upstream of the region encoding the LPXTG motif and was found in all CC1 strains except the strain HKU09-01. The size of the SlsG predicted protein sequences, varying from 1604 to 2286 amino acids, correlated with the Rib repeat number ranging from 7 to 21. A frameshift was located within Rib repeats for VISLISI_21 and VISLISI_25 isolates. On the other hand, SlsB, SlsC and SlsF proteins were highly conserved (99–100% of identity) in the 21 *S. lugdunensis* strains.

Of note, notable diversity was observed in *lug locus* encoding a peptide antibiotic (lugdunin). Of the 21 strains, 8 contained a frameshift in one to 2 genes of the *locus* while the latter was absent in 2 strains.

### Description of the CC-Dependent Genetic Diversity of the *S. lugdunensis ess Locus*

**FIGURE 2:**
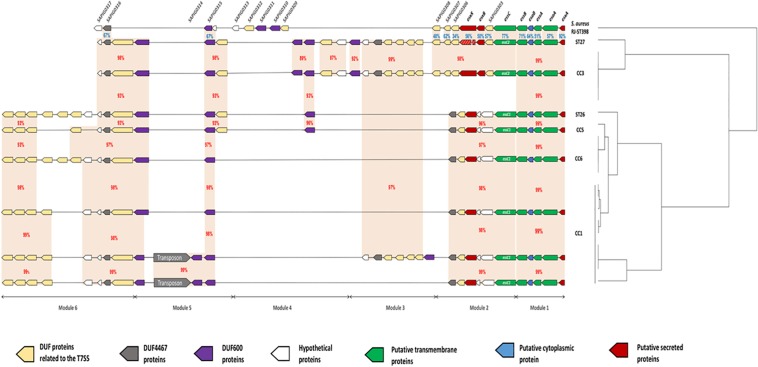
Schematic representation of 8 genetic organizations of the *ess* locus from genomes of 21 strains of *S. lugdunensis.* For each strain, clonal complexes (CC) are indicated to the right. The *ess* locus was divided into 6 modules depending on gene-based content and relative conservation defined with the Artemis comparison tool. Module 1 contained 5 genes homologous of the core components (*esxA* to *essB*) of *S. aureus* T7SS. Module 2 included the *essC gene*, of which there are 2 variants, each associated with a distinct cluster of 5–6 genes. Modules 3, 4 and 6 contained a complex arrangement of coding sequences that encode a variable number of uncharacterized proteins. Module 5 is composed of 2 distinct genetic organizations: one of them is composed of a transposon associated with 3 coding sequences encoding hypothetical DUF600 proteins whereas the other one is comprised of only 2 genes. Orange areas highlight conserved coding sequences between strains with the percentage of amino acid identity in red between *S. lugdunensis* strains and in blue with *S. aureus* strain RJ-ST398.

Comparative genomic analysis showed variations in the size of the *ess locus* of the 21 *S. lugdunensis* isolates. Indeed, *ess locus* size ranged from 22134 bp (HKU09-01) to 38834 bp (SL29 and SL122). This difference was the result of an important polymorphism identified in CC1 strains. For a better understanding of these variations, we divided the *ess locus* into 6 distinct modules, each based on gene content and relative conservation (Figure 2). Eight genetic organizations were observed.

Module 1 contained five genes present in all sequences analyzed and homologous to genes encoding proteins of T7SS in *S. aureus*: EsxA, EsaA, EssA, EsaB and EssB. This module was highly conserved among all *S. lugdunensis* strains, with slight modifications in the amino acid sequences of these five proteins (99–100% identity).

**FIGURE 3:**
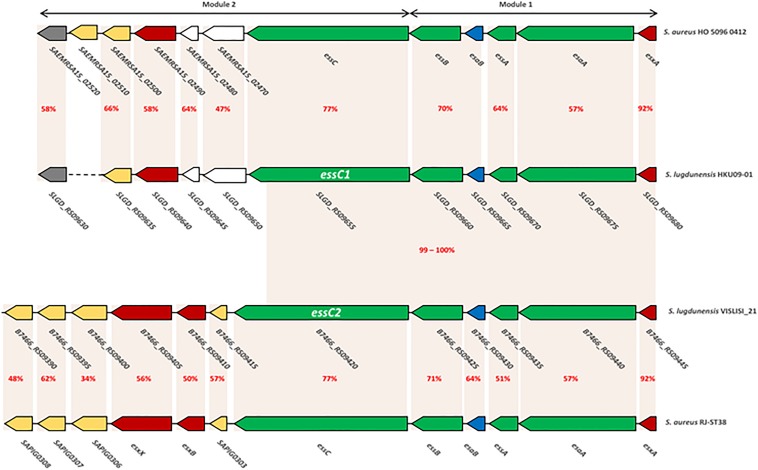
Comparison of *ess* modules 1 and 2 of *S. aureus* HO 5096 0412 and RJ-ST398 strains with HKU09-01 and VISLISI_21 *S. lugdunensis* strains. Gene-based content and relative conservation defined with the Artemis comparison tool. Orange areas highlight conserved coding sequences between strains with the percentage of amino acid identity in red.

Module 2 was variable between *S. lugdunensis* CCs. Indeed, 2 distinct organizations of the *ess locus* were identified: one shared by all CC3 strains and by one CC7 strain (C33), and the other one exhibited by other CC strains (CC1, CC5 and CC6). The first important variation concerned the *essC* gene for which 2 *essC* sequence variants (*essC1* and *essC2*) were observed. The 5′ region of the *essC* gene was conserved within all *S. lugdunensis* isolates (3762 bp) whereas the 3′ region (around 681 bp) differed according to CCs. The *essC1* variant, identified in 15 strains, encodes a putative protein of 1476 residues whereas the *essC2* variant encodes a putative protein of 1481 amino acids. This latter was only identified in 5 CC3 strains and in one CC7 strain (C33). Of note, *S. lugdunensis* N920143 contained a frameshift in the gene encoding the membrane-associated protein EssC. Otherwise, each *essC* gene variant was associated with its own cluster of genes. *essC1* gene was associated with five genes highly conserved among 15 *S. lugdunensis* isolates while *essC2* gene variant was associated with six genes conserved for six isolates. Conversely, these six genes shared no identity with the five genes associated with the *essC1* variant (Figure 3).

In modules 3, 4 and 6, a complex arrangement of sequences encoding a variable number of uncharacterized proteins was observed. In particular, an important number of genes which encode proteins containing Domains of Unknown Function (DUFs) were identified. Among them, DUF600 and some others (DUF5079, DUF5080, DUF5020, DUF5084 and DUF5085) are suspected to be involved in T7SS on the basis of homology with characterized T7SS systems. The number of genes encoding DUF600 proteins varied from 2 in CC6 strains to 5 in CC3 strains while the number of genes encoding the other DUF proteins ranged from 6 to 11 among all strains (Figure 2).

In module 5, a transposon was found in 4 of the 9 CC1 isolates, associated with genes encoding hypothetical DUF600 proteins (Figure 2).

Here, the comparison focuses on modules 1 and 2 which are the only ones to have a strong homology with those of *S. aureus* (48–92% identity). This was performed on the one hand between the genomes of *S. aureus* HO 5096 0412 and *S. lugdunensis* HKU09-01 strains, and on the other hand between the genomes of *S. aureus* RJ-ST398 and *S. lugdunensis* VISLISI_21 strains. These 4 strains were chosen to be representative of the diversity of the *locus* in both species (Figure 3).

Comparative analysis of module 1 showed that *S. lugdunensis* genes *SLGD_RS09680* (HKU09-01) and *B7466_RS09445* (VISLISI_21) which encode a putative secreted protein shared 92% identity with the EsxA protein of *S. aureus* strains. This putative protein of 97 amino acids contains a WXG100 domain and a conserved C-terminal hydrophobic pattern for the formation of specific α-helical surface. Moreover, several putative transmembrane proteins in *S. lugdunensis* HKU09-01 and VISLISI_21 isolates showed homologies (51–64% identity) with the transmembrane proteins EsaA, EssA and EssB of *S. aureus* strains (Tables 6, 7). The EsaB cytoplasmic protein of *S. aureus* strains exhibited 64% identity with proteins SLGD_RS09065 (HKU09-01) and B7466_RS09420 (VISLISI_21) which contained a YukD family domain. Thus, functional analysis of T7SS core components using pfam database allowed the prediction and identification of the same protein domains for *S. aureus* and *S. lugdunensis*.

**TABLE 6:** Comparison of proteins encoded by *ess* modules 1 and 2 of *S. aureus* HO 5096 0412 with HKU09-01 *S. lugdunensis* strains.

***S. aureus* HO 5096 0412 strain**			***S. lugdunensis* HKU09-01 strain**			
	
**Proteins**	**Length (amino acids)**	**pfam family domain**	**Proteins**	**Length (amino acids)**	**pfam family domain**	**% identity**
EsxA	97	WXG100	SLGD_RS09680	97	WXG100	92%
EsaA	1009	N/A	SLGD_RS09675	1006	N/A	57%
EssA	153	EssA	SLGD_RS09670	144	EssA	64%
EsaB	80	YukD	SLGD_RS09665	80	YukD	70%
EssB	444	YukC	SLGD_RS09660	437	YukC	70%
EssC	1476	FtsK SpoIIIE N two FtsK SpoIIIE	SLGD_RS09655	1476	FtsK SpoIIIE N two FtsK SpoIIIE	77%
SAEMRSA15_02470	359	N/A	SLGD_RS09650	360	N/A	47%
SAEMRSA15_02480	96	N/A	SLGD_RS09645	96	N/A	64%
SAEMRSA15_02490	420	LXG	SLGD_RS09640	420	LXG	58%
SAEMRSA15_02500	137	DUF5082	SLGD_RS09635	137	DUF5082	66%
SAEMRSA15_02510	157	DUF5083	N/A	N/A	N/A	N/A
SAEMRSA15_02520	124	DUF4467	SLGD_RS09630	127	DUF4467	58%

**TABLE 7:** Comparison of proteins encoded by *ess* modules 1 and 2 of *S. aureus* RJ-ST398 strains with VISLISI_21 *S. lugdunensis* strains.

***S. aureus* RJ-ST398 strain**			***S. lugdunensis* VISLISI_21 strain**			
	
**Proteins**	**Length (amino acids)**	**pfam family domain**	**Proteins**	**Length (amino acids)**	**pfam family domain**	**% identity**
EsxA	97	WXG100	B7466_RS09445	97	WXG100	92%
EsaA	1009	N/A	B7466_RS09440	1006	N/A	57%
EssA	153	EssA	B7466_RS09435	144	EssA	51%
EsaB	80	YukD	B7466_RS09430	80	YukD	64%
EssB	444	YukC	B7466_RS09425	437	YukC	71%
EssC	1481	FtsK SpoIIIE N two FtsK SpoIIIE	B7466_RS09420	1481	FtsK SpoIIIE N two FtsK SpoIIIE	77%
SAPIG0303	98	DUF5344	B7466_RS09415	95	DUF5344	57%
EsxB	134	DUF5082	B7466_RS09410	107	DUF5082	50%
EsxX	517	LXG	B7466_RS09405	523	LXG	56%
SAPIG0306	228	DUF5079	B7466_RS09400	223	DUF5079	34%
SAPIG0307	149	DUF5085	B7466_RS09395	150	DUF5085	62%
SAPIG0308	149	DUF5085	B7466_RS09390	151	DUF5085	48%

Then, pairwise comparison of module 2 showed homologous genes and comparable organization (Figure 3 and Tables 6, 7). First, the *essC1* and *essC2* variants of *S. lugdunensis* strains encode proteins sharing 77% identity with the EssC proteins of *S. aureus* strains. The putative EssC proteins of *S. lugdunensis* contain a N-terminal FtsK-SpoIIIE domain for DNA transportation and 2 domains of the SpoIIIE-FtsK-like ATPase family, like those of *S. aureus*. Second, in both *S. aureus* and *S. lugdunensis*, variants of the *essC* gene are associated with their own gene cluster. Among putative encoded proteins, there are proteins with a N-terminal LXG domain or a central WXG motif (Tables 6, 7). For example, the putative proteins B7466_RS09410 and B7466_RS09405 of *S. lugdunensis* VISLISI 21 have 50% and 56% identity with the EsxB and EsxX proteins of *S. aureus* RJ-ST398, respectively.

## Discussion

*Staphylococcus lugdunensis* is associated with a wide range of diseases including skin and soft tissue infections, infective endocarditis and bone and joint infections (Frank et al., 2008). This organism is considered as an opportunistic pathogen which can cause infections with unusual severity compared to other CoNS. This particular virulence is not currently explained by the rare virulence factors described so far, and no typing method (Chassain et al., 2012; Didi et al., 2014) has yet been able to identify invasive clonal complexes as described for *Neisseria meningitidis* (Bratcher et al., 2012) or *Streptococcus pneumoniae* (Dagerhamn et al., 2008).

The advent of high throughput sequencing technology offers new insights into the identification of determinants associated with bacterial virulence. Before this study, only 15 complete genomes of *S. lugdunensis* were available in GenBank and no comparison between pathogenic and carriage strains had been conducted. In this context, we sequenced the genome of 6 new strains (3 pathogenic and 3 carriage strains) whose virulence was characterized in a *G. mellonella* infection model.

This model has been applied to characterize the virulence and pathogenesis of a wide range of microorganisms (Kavanagh and Reeves, 2004; Ramarao et al., 2012) but has never been implemented for *S. lugdunensis*. Interestingly, our results suggest that this model could be useful for assessing the virulence of *S. lugdunensis in vivo* as the SSTI-associated strain (SL55) and the infective endocarditis strain (SL13) were significantly more lethal than the four other isolates. Although insects lack an adaptative immune response, their innate immune response still retains remarkable similarities with the immune response in vertebrates; this allowed to obtain relevant information about the infection process (Kavanagh and Reeves, 2004). In addition, infected larvae can be incubated at 37°C, which favors the study of human pathogenic bacteria (Kavanagh and Reeves, 2004). Of course, these preliminary results must be confirmed in a larger collection of isolates. Although it will not replace mammalian models, the larvae of *G. mellonella* provide a fast and cost-effective alternative to collect initial data.

With the addition of six new *S. lugdunensis* genomes, this study has raised the total number of complete genomes available for comparative purposes from 15 to 21. This result has increased the genetic diversity of the genomic data for this species as these strains were collected from one Swedish and 3 different French cities, whilst 7 of the 15 genomes in GenBank originated from a unique French location (Argemi et al., 2018).

Here, we have characterized the pan-, core- and accessory-genome of the species, particularly according to CCs defined by MLST. Furthermore, we have described the first genomic comparison of carriage and pathogenic strains representative of the major lineages of *S. lugdunensis* to explore whether virulence can be linked to specific genes or genetic variations.

### Presence of MGE Despite a Closed Pan-Genome

Comparative genomic analysis favored a closed pan-genome, as suggested by Heaps’ law of extrapolation applied by Argemi et al. (2018). The *S. lugdunensis* pan-genome appeared in fact to be poorly expanding with the inclusion of six new complete genomes reaching a total of only 3077 genes. On the contrary, *S. aureus* and *S. epidermidis* pan-genomes reached more than 3800 genes, with a constant increase when adding new genomes (Argemi et al., 2018). This should be confirmed by the analysis of human *S. lugdunensis* strains from other continents but also of environmental and animal origin.

In this context, it is not surprising that a large number of *S. lugdunensis* strains have barriers to horizontal gene transfer like CRISPR/Cas system or RMS (Argemi et al., 2018). Our study shows that 48% of strains possess a CRISPR/Cas system. These findings confirm the particular status of this species among CoNS. Indeed, Rossi et al. (2017) showed that only 9% (i.e., 3/34) and 3% (i.e., 1/32) of *S. epidermidis* and *Staphylococcus haemolyticus* strains respectively presented characteristic CRISPR/Cas systems. Of note, the CRISPR/Cas system was only found in CC1 strains. This high prevalence may be explained by the fact that most of the sequenced genomes belonged to CC1 strains (9/21, i.e., 43%). This CC is currently over-represented in NCBI in comparison with other CCs (CC3 [5/21, i.e., 24%], CC5 [1/21, i.e., 4%], CC6 [4/21, i.e., 19%] and CC7 [2/21, i.e., 10%]) in relation to its global distribution (Yeh et al., 2016; Dahyot et al., 2019). It would be interesting to sequence the whole genome of CC2 and CC4 strains, as none are available in the GenBank database.

Despite the presence of CRISPR/Cas immunity systems and several type I and type II RMS, most (15/21, i.e., 71%) of the *S. lugdunensis* strains have acquired MGE probably from other staphylococcal species by horizontal gene transfer. This was particularly observed for CC1 strains. This latter could be explained by a frameshift within the *csm6* gene that might impact the initial role of the CRISPR/Cas system. Indeed, in *S. epidermidis*, the protein Csm6 was found essential for preventing plasmid transformation (Hatoum-Aslan et al., 2014; Jiang et al., 2016). Further investigations in a larger collection of *S. lugdunensis* strains and functional characterization are needed to understand the ability of CRISPR/Cas to prevent MGE acquisition. Another hypothesis would be that MGE may have been present before acquisition of the CRISPR/Cas region. Enriching the database with genomes of ancestral strains would help to resolve this issue.

On the contrary, while CC3 strains do not exhibit CRISPR/Cas systems or RMS, only one plasmid was detected within their genome. This can be related to the great difficulties encountered in genetically manipulating *S. lugdunensis*. The rare mutant strains published were only constructed after the introduction of plasmids by protoplast transformation (Szabados et al., 2011; Marlinghaus et al., 2012) or by electroporation of *Escherichia coli* strains expressing *hsdM*/*hsdS* RMS genes of *S. lugdunensis*. Interestingly, despite a new electroporation protocol, Heilbronner et al. (2013) highlighted that CC3 and CC4 isolates were harder to transform than CC1, CC2 and CC5 strains. This suggests that other mechanisms may play the role of barrier against the acquisition of MGE in *S. lugdunensis*. Bacteria can display physical barriers such as extracellular polysaccharidic substances, including capsules to prevent phage infection (Wilkinson and Holmes, 1979; Labrie et al., 2010). Thus, *cap8* could be involved in the synthesis of capsule in *S. lugdunensis* CC3 strains and act as a protective barrier.

*S. lugdunensis* exhibits a highly conserved antibiotic susceptibility to most anti-staphylococcal antibiotics, which may be related to these different barriers against the integration of foreign DNA (Higaki et al., 1999; McHardy et al., 2017). Indeed, only one plasmid carrying an antibiotic resistance gene (*tetK*) was identified in a newly sequenced genome. Fosfomycin, penicillin and fusidic acid resistance-associated genes were identified with a chromosomic location. Fosfomycin resistance-associated gene was highly conserved among the 21 strains while the distribution of penicillin and fusidic acid resistance genes was variable between isolates. This finding is in accordance with the results of McHardy et al. (2017). who showed that the *blaZ* gene was detected in 39.1% of oxacillin-susceptible *S. lugdunensis* isolates. The presence of the *blaZ gene* was not correlated with CCs.

In this study, no specific virulence determinant was identified in *S. lugdunensis* pathogenic isolates compared to carriage strains. Nevertheless, two genomic islands were only identified in three pathogenic strains. One genomic island of 16 kb carries fusidic acid resistance *fusB* gene and *vapE* gene which might contribute to bacterial adaptation and virulence (Novick et al., 2010; Chen et al., 2013). A second one of 13 kb, partially homologous to the pathogenicity islands of *S. aureus* (SaPI) (Novick and Subedi, 2007), was found in *S. lugdunensis* strains SL13 (causing infective endocarditis) and VISLISI_33 (causing liver abscess). In *S. aureus*, SaPIs play an important role in genomic evolution, leading to an increase in its ability to cause severe infections by acquisition of numerous virulence determinants such as pore-forming toxins and superantigens (Malachowa and DeLeo, 2010; Novick and Ram, 2017). In *S. lugdunensis*, no enterotoxin, superantigen or toxic shock syndrome toxin-1 was found in this region. However, an Ear penicillin binding protein and a FhuD ferrichrome ABC transporter were carried by this potential pathogenicity island. *S. aureus* Ear protein may act as a superantigenic exoprotein which could play a role in virulence (Singh et al., 2017). Otherwise, the ferric hydroxamate uptake D (FhuD) protein could be involved in iron acquisition by *S. lugdunensis* as it can promote ferrisiderophore import in *S. aureus* (Beasley et al., 2009) and facilitate heme uptake in blood and serum (Malachowa et al., 2011). Therefore, identification of these genes in the genomes of *S. lugdunensis* suggests that genomic island, other than MGE as bacteriophage or plasmids, could also contribute to the acquisition of antibiotic resistance and virulence genes in this species even if the phenomenon is probably rare (Argemi et al., 2018).

### CC-Dependent Genetic Variations of Putative Virulence-Associated *loci*

Based on genomic comparison using VFDB, we identified CC-dependent polymorphisms in several *loci* (*agr, cap, sls* and *ess*) putatively associated with *S. lugdunensis* virulence.

Our study shows that all CC3 strains belonged to the *agrSl-*type II whereas all other strains were associated with the allele *agrSl-*type I. However, no link has been shown between *S. lugdunensis agr* polymorphisms (*agrSl-*types) and specific clinical infection entities. Many studies have attempted to associate *agr* types with one or more of the biological characteristics of staphylococcal species. Tseng et al. (2015) thus showed that 7 of 11 isolates collected from bacteremia belonging to *agrSl-*type II presented higher hemolytic and protease activities than 4 *agrSl-*type I isolates. In *S. aureus*, *agr* groups have been shown to vary by clonal lineage distribution, antibiotic resistance profile, biofilm formation, expression of virulence factors, and autoinducing peptide structures (Holtfreter et al., 2007; Tan et al., 2018). In addition, Jarraud et al. (2000) have shown that *S. aureus* endocarditis strains were mainly associated with phylogenetic group AF2 (*agr* groups I or II) while phylogenetic group AF1 (*agr* group IV) strains are mainly responsible for generalized exfoliative syndromes and bullous impetigo. Similarly, *agr*-specificity groups in *S. epidermidis* are also associated with specific clinical infections (Hellmark et al., 2013; Harris et al., 2017). The potential impact of this CC-*agrSl-*type association on the regulation of virulence could be explored by a functional analysis of the *agr locus* in *S. lugdunensis*.

Interestingly, CC3 strains are also distinguished from all other strains by the allele of the *cap* gene (*cap8*). Except for the study of Lambe et al. (1994) that showed that *S. lugdunensis* possessed glycocalyx, no work has yet described the prevalence, composition, diversity and role of the capsule in this species. In *S. aureus*, several studies have shown that serotype 5 or 8 capsular isolates account for approximately 80% of isolates recovered from humans, the remaining isolates being not encapsulated or non-typeable (Arbeit et al., 1984; Roghmann et al., 2005; Cocchiaro et al., 2006). Like *S. aureus*, the genes specifying capsular polysaccharides in *S. lugdunensis* are encoded by a highly conserved *cap locus* including 4 genes, *capHIJK*, which may specify chemical diversity among serotypes (O’Riordan and Lee, 2004).

Heilbronner et al. (2011) described 7 genes encoding “*S. lugdunensis* surface proteins” named SlsA to SlsG within the genome of *S. lugdunensis* N920143 strain. Here, we have shown that several Sls proteins were absent or potentially non-functional in some *S. lugdunensis* strains according to CC. These CC-dependent differences could result in variations in the biofilm-forming and adhesion capacities between isolates. Indeed, SlsA contains putative protein domains and structural organization homologous to a known cell wall-associated biofilm Bhp protein (Post et al., 2017), which has been shown to promote primary attachment to abiotic surfaces as well as intercellular adhesion during biofilm formation (Cucarella et al., 2001; Bowden et al., 2005). In addition, the SlsD protein has putative structural similarity with members of the Sdr adhesin family such as SdrF and SdrG, crucial for the attachment of *S. epidermidis* to surfaces coated with human fibrinogen or type I collagen (Bowden et al., 2005; Arrecubieta et al., 2007). A functional characterization of these Sls proteins could be helpful to explore whether they are involved in the ability of *S. lugdunensis* to bind medical devices or biotic surfaces, such as host tissues.

On the other hand, significant genetic variations independent of CCs were detected in the *lug locus*. The impact of this diversity on the production and activity of lugdunine as well as on the structure of the microbiota of ecological niches of *S. lugdunensis* remains to be demonstrated (Zipperer et al., 2016).

### High Genetic Diversity of the *ess*/Type VII *Locus*, a Potential Key Virulence Determinant

This study provides, to the best of our knowledge, the first description of the CC-dependent genetic organization of the *ess locus* from *S. lugdunensis*. Initially mentioned for one strain of *S. lugdunensis* (Warne et al., 2016), this *locus* has shown considerable diversity for a clonal species (eight genetic CC-dependent organizations among only 21 strains). Homologous genes of those encoding the six core components of *S. aureus* T7SS were identified among the 21 genomes: the four membrane-associated proteins (EsaA, EssA, EssB, and EssC), a soluble cytosolic protein (EsaB) and the Early Secreted Antigenic Target-6 kDa (ESAT-6, also termed EsxA) (Warne et al., 2016; Unnikrishnan et al., 2017). Membrane and cytoplasmic proteins have been demonstrated to be necessary to export effector proteins across the bacterial membrane during host infection (Unnikrishnan et al., 2017), as proteins from the superfamily WXG100 (Burts et al., 2005) or LXG domain-containing proteins (Dai et al., 2017).

Whereas *S. aureus* T7SS can secrete two WXG100 proteins (EsxA, EsxB), only one member of the WXG100 protein superfamily, EsxA, has been identified in *S. lugdunensis*. As for *S. aureus* EsxA, the putative *S. lugdunensis* EsxA protein contains conserved C-terminal hydrophobic patterns HxxxD/ExxhxxxH (where H stands for highly conserved hydrophobic, h for less conserved hydrophobic residues, x for any amino acid and D/E for either aspartic or glutamic acids, respectively). This domain could constitute a key component of T7SS-substrate recognition (Poulsen et al., 2014).

In addition, *S. lugdunensis* possesses a gene encoding a LXG domain-containing protein which is found in a group of polymorphic toxins predicted to use the T7SS pathway (Zhang et al., 2011; Whitney et al., 2017). Thereby, these data highlight the possible extracellular translocation of these proteins. In *S. aureus*, proteins with domain LXG are involved in immune evasion and virulence as the protein EsxX (Dai et al., 2017) or in bacterial competition as the EsaD nuclease (Anderson et al., 2013; Cao et al., 2016). Of note, no gene homologous to the *esaD* gene encoding the anti-bacterial toxin with a C-terminal nuclease domain was identified within the genome of *S. lugdunensis* strains.

A great diversity in the genetic organization of the *ess locus* was described by Warne et al. (2016) in 153 *S. aureus* isolates belonging to different CCs. They showed that the *S. aureus essC* gene exhibited 4 sequence variants, each associated with a specific cluster of 4–6 different genes. In *S. aureus*, the *essC4* variant (homologous to the *S. lugdunensis essC1* variant) was only found in CC22 isolates whereas the *essC2* variant (homologous to the *S. lugdunensis essC2* variant) was identified in CC15 and ST398 strains. In *S. lugdunensis*, a strong association between CCs and *essC* variants was also observed. These results suggest that *S. lugdunensis* strains may have acquired these regions independently and from difference sources.

Moreover, genetic diversity was observed downstream of modules 1 and 2, based on (i) a variable number of genes encoding uncharacterized proteins, (ii) the presence of a cluster of 7 genes specific to CC1 and CC3, (iii) the insertion of a transposon in CC1 strains, and (iv) the variable copy number of genes encoding DUF600 proteins. The mechanisms of occurrence of these inter- and intra-CC variations are unclear and need further investigations in a larger collection of *S. lugdunensis* strains, and especially in strains belonging to CC2 and CC4 which could present different organizations of this *locus*.

Thus, we describe here homologs of a large number of genes encoding T7SS in *S. lugdunensis* which shares a high number of clinical features with *S. aureus*, including ability to form abscesses. *S. lugdunensis* is the sole CoNS to possess all the components required for functional Ess machinery. T7SS could play a key role in abscess development and hence in the currently poorly understood virulence of this bacterial species.

Taken together, these results support the “closed” state of the pan-genome of *S. lugdunensis*, but identification of MGE carrying putative virulence and resistance genes suggests some possible horizontal gene transfers between *S. lugdunensis* and other staphylococcal species. No specific variation or virulence determinant was associated with the pathogenic strains, but the distribution of potential genomic islands constitutes an interesting option to explore in a larger collection of strains. Here, we identified several CC-dependent variations, especially for CC3 strains which showed specific variations in several *loci* potentially associated with virulence. Further phenotypic and functional studies are needed to fully characterize this particular CC and to evaluate the role of T7SS in the virulence of *S. lugdunensis*. Finally, whole genome sequencing of CC2 and CC4 strains is needed for a complete picture of the genetic diversity of *S. lugdunensis*.

## Data Availability Statement

The datasets generated for this study can be found in the GenBank database, accession: CP041722, CP041723, CP041724, CP041725, CP041726, CP041727.

## Author Contributions

JL, SDa, PF, and MP-C designed the study. JL, SDi, and AP analyzed the data. J-CG and MA performed the *Galleria mellonella* infection experiments. JL, SDa, and MP-C wrote the manuscript. All authors have read and approved the final version of the manuscript.

## Conflict of Interest

The authors declare that the research was conducted in the absence of any commercial or financial relationships that could be construed as a potential conflict of interest.

## Footnotes

1 http://bigsdb.web.pasteur.fr/staphlugdunensis

2 https://git.facom.ufms.br/bioinfo/orthologsorter

3 https://bigsdb.pasteur.fr/staphlugdunensis/

4 http://phaster.ca

5 http://www.mgc.ac.cn/VFs

6 https://cge.cbs.dtu.dk/services/ResFinder

## References

[B1] Anderson M. Aly K. A. Chen Y.-H. Missiakas D. (2013). Secretion of atypical protein substrates by the ESAT-6 secretion system of *Staphylococcus aureus*: ESAT-6 secretion in *S. aureus*. *Mol. Microbiol.* 90 734–743. 10.1111/mmi.12395 24033479 PMC3951145

[B2] Anguera I. (2005). *Staphylococcus lugdunensis* infective endocarditis: description of 10 cases and analysis of native valve, prosthetic valve, and pacemaker lead endocarditis clinical profiles. *Heart* 91:e10. 10.1136/hrt.2004.040659 15657200 PMC1768720

[B3] Arbeit R. D. Karakawa W. W. Vann W. F. Robbins J. B. (1984). Predominance of two newly described capsular polysaccharide types among clinical isolates of *Staphylococcus aureus*. *Diagn. Microbiol. Infect. Dis.* 2 85–91. 10.1016/0732-8893(84)90002-6 6232086

[B4] Argemi X. Dahyot S. Lebeurre J. Hansmann Y. Ronde Oustau C. Prévost G. (2017a). *Staphylococcus lugdunensis* small colony variant conversion resulting in chronic prosthetic joint infection. *Méd. Mal. Infect.* 47 498–501. 10.1016/j.medmal.2017.05.005 28943172

[B5] Argemi X. Martin V. Loux V. Dahyot S. Lebeurre J. Guffroy A. (2017b). Whole genome sequencing of 7 strains of *Staphylococcus lugdunensis* allows identification of mobile genetic elements. *Genome Biol. Evol.* 9:77. 10.1093/gbe/evx077 28444231 PMC5425232

[B6] Argemi X. Matelska D. Ginalski K. Riegel P. Hansmann Y. Bloom J. (2018). Comparative genomic analysis of *Staphylococcus lugdunensis* shows a closed pan-genome and multiple barriers to horizontal gene transfer. *BMC Genom.* 19:621. 10.1186/s12864-018-4978-1 30126366 PMC6102843

[B7] Arias M. Tena D. Apellániz M. Asensio M. P. Caballero P. Hernández C. (2010). Skin and soft tissue infections caused by *Staphylococcus lugdunensis*: report of 20 cases. *Scand. J. Infect. Dis.* 42 879–884. 10.3109/00365548.2010.509332 20735327

[B8] Arndt D. Grant J. R. Marcu A. Sajed T. Pon A. Liang Y. (2016). PHASTER: a better, faster version of the PHAST phage search tool. *Nucleic Acids Res.* 44 W16–W21. 10.1093/nar/gkw387 27141966 PMC4987931

[B9] Arrecubieta C. Lee M.-H. Macey A. Foster T. J. Lowy F. D. (2007). SdrF, a *Staphylococcus epidermidis* surface protein, binds type I collagen. *J. Biol. Chem.* 282 18767–18776. 10.1074/jbc.M610940200 17472965

[B10] Beasley F. C. Vinés E. D. Grigg J. C. Zheng Q. Liu S. Lajoie G. A. (2009). Characterization of staphyloferrin a biosynthetic and transport mutants in *Staphylococcus aureus*. *Mol. Microbiol.* 72 947–963. 10.1111/j.1365-2958.2009.06698.x 19400778

[B11] Bieber L. Kahlmeter G. (2010). *Staphylococcus lugdunensis* in several niches of the normal skin flora. *Clin. Microbiol. Infect.* 16 385–388. 10.1111/j.1469-0691.2009.02813.x 19519842

[B12] Bocher S. Tonning B. Skov R. L. Prag J. (2009). *Staphylococcus lugdunensis*, a common cause of skin and soft tissue infections in the community. *J. Clin. Microbiol.* 47 946–950. 10.1128/JCM.01024-08 19244465 PMC2668335

[B13] Bowden M. G. Chen W. Singvall J. Xu Y. Peacock S. J. Valtulina V. (2005). Identification and preliminary characterization of cell-wall-anchored proteins of *Staphylococcus epidermidis*. *Microbiol. Read. Engl.* 151 1453–1464. 10.1099/mic.0.27534-0 15870455

[B14] Bratcher H. B. Bennett J. S. Maiden M. C. J. (2012). Evolutionary and genomic insights into meningococcal biology. *Future Microbiol.* 7 873–885. 10.2217/fmb.12.62 22827308 PMC3492750

[B15] Burts M. L. DeDent A. C. Missiakas D. M. (2008). EsaC substrate for the ESAT-6 secretion pathway and its role in persistent infections of *Staphylococcus aureus*. *Mol. Microbiol.* 69 736–746. 10.1111/j.1365-2958.2008.06324.x 18554323 PMC2597432

[B16] Burts M. L. Williams W. A. DeBord K. Missiakas D. M. (2005). EsxA and EsxB are secreted by an ESAT-6-like system that is required for the pathogenesis of *Staphylococcus aureus* infections. *Proc. Natl. Acad. Sci. U. S. A.* 102 1169–1174. 10.1073/pnas.0405620102 15657139 PMC545836

[B17] Cao Z. Casabona M. G. Kneuper H. Chalmers J. D. Palmer T. (2016). The type VII secretion system of *Staphylococcus aureus* secretes a nuclease toxin that targets competitor bacteria. *Nat. Microbiol.* 2:16183. 10.1038/nmicrobiol.2016.183 27723728 PMC5325307

[B18] Carver T. J. Rutherford K. M. Berriman M. Rajandream M.-A. Barrell B. G. Parkhill J. (2005). ACT: the artemis comparison tool. *Bioinform. Oxf. Engl.* 21 3422–3423. 10.1093/bioinformatics/bti553 15976072

[B19] Chassain B. Lemee L. Didi J. Thiberge J.-M. Brisse S. Pons J.-L. (2012). Multilocus sequence typing analysis of *staphylococcus lugdunensis* implies a clonal population structure. *J. Clin. Microbiol.* 50 3003–3009. 10.1128/JCM.00988-12 22785196 PMC3421835

[B20] Chen H.-J. Chang Y.-C. Tsai J.-C. Hung W.-C. Lin Y.-T. You S.-J. (2013). New structure of phage-related islands carrying *fusb* and a virulence gene in fusidic acid-resistant *Staphylococcus epidermidis*. *Antimicrob. Agents Chemother.* 57 5737–5739. 10.1128/AAC.01433-13 23979742 PMC3811319

[B21] Chen L. Zheng D. Liu B. Yang J. Jin Q. (2016). VFDB 2016: hierarchical and refined dataset for big data analysis—10 years on. *Nucleic Acids Res.* 44 D694–D697. 10.1093/nar/gkv1239 26578559 PMC4702877

[B22] Cocchiaro J. L. Gomez M. I. Risley A. Solinga R. Sordelli D. O. Lee J. C. (2006). Molecular characterization of the capsule locus from non-typeable *Staphylococcus aureus*. *Mol. Microbiol.* 59 948–960. 10.1111/j.1365-2958.2005.04978.x 16420363

[B23] Cucarella C. Solano C. Valle J. Amorena B. Lasa I. Penadés J. R. (2001). Bap, a *Staphylococcus aureus* surface protein involved in biofilm formation. *J. Bacteriol.* 183 2888–2896. 10.1128/JB.183.9.2888-2896.2001 11292810 PMC99507

[B24] Dagerhamn J. Blomberg C. Browall S. Sjöström K. Morfeldt E. Henriques-Normark B. (2008). Determination of accessory gene patterns predicts the same relatedness among strains of *Streptococcus pneumoniae* as sequencing of housekeeping genes does and represents a novel approach in molecular epidemiology. *J. Clin. Microbiol.* 46 863–868. 10.1128/JCM.01438-07 18160453 PMC2268355

[B25] Dahyot S. Lebeurre J. Argemi X. François P. Lemée L. Prévost G. (2018). Multiple-locus variable number tandem repeat analysis (MLVA) and tandem repeat sequence typing (TRST), helpful tools for subtyping *Staphylococcus lugdunensis*. *Sci. Rep.* 8:11669. 10.1038/s41598-018-30144-y 30076395 PMC6076266

[B26] Dahyot S. Lebeurre J. Laumay F. Argemi X. Dubos C. Lemée L. (2019). *fbl*-typing of *Staphylococcus lugdunensis*: a frontline tool for epidemiological studies, but not predictive of fibrinogen binding ability. *Front. Microbiol.* 10:1109. 10.3389/fmicb.2019.01109 31156610 PMC6533592

[B27] Dai Y. Wang Y. Liu Q. Gao Q. Lu H. Meng H. (2017). A Novel ESAT-6 secretion system-secreted protein esxx of community-associated *Staphylococcus aureus* lineage ST398 contributes to immune evasion and virulence. *Front. Microbiol.* 8:819. 10.3389/fmicb.2017.00819 28529509 PMC5418362

[B28] Didi J. Lemee L. Gibert L. Pons J.-L. Pestel-Caron M. (2014). Multi-virulence-locus sequence typing of *Staphylococcus lugdunensis* generates results consistent with a clonal population structure and is reliable for epidemiological typing. *J. Clin. Microbiol.* 52 3624–3632. 10.1128/JCM.01370-14 25078912 PMC4187764

[B29] Douiri N. Hansmann Y. Lefebvre N. Riegel P. Martin M. Baldeyrou M. (2016). *Staphylococcus lugdunensis?*: a virulent pathogen causing bone and joint infections. *Clin. Microbiol. Infect.* 22 747–748. 10.1016/j.cmi.2016.05.031 27297318

[B30] Dufour P. Jarraud S. Vandenesch F. Greenland T. Novick R. P. Bes M. (2002). High Genetic Variability of the *agr* locus in *Staphylococcus* Species. *J. Bacteriol.* 184 1180–1186. 10.1128/jb.184.4.1180-1186.2002 11807079 PMC134794

[B31] Ebright J. R. Penugonda N. Brown W. (2004). Clinical experience with *Staphylococcus lugdunensis* bacteremia: a retrospective analysis. *Diagn. Microbiol. Infect. Dis.* 48 17–21. 10.1016/j.diagmicrobio.2003.08.008 14761717

[B32] Frank K. L. del Pozo J. L. Patel R. (2008). From clinical microbiology to infection pathogenesis: how daring to be different works for *Staphylococcus lugdunensis*. *Clin. Microbiol. Rev.* 21 111–133. 10.1128/CMR.00036-07 18202439 PMC2223846

[B33] Gupta P. K. (2008). Single-molecule DNA sequencing technologies for future genomics research. *Trends Biotechnol.* 26 602–611. 10.1016/j.tibtech.2008.07.003 18722683

[B34] Harris L. G. Dudley E. Rohde H. Frommelt L. Siemssen N. Wilkinson T. S. (2017). Limitations in the use of PSMγ, *agr*, RNAIII, and biofilm formation as biomarkers to define invasive *Staphylococcus epidermidis* from chronic biomedical device-associated infections. *Int. J. Med. Microbiol. IJMM* 307 382–387. 10.1016/j.ijmm.2017.08.003 28826573

[B35] Hatoum-Aslan A. Maniv I. Samai P. Marraffini L. A. (2014). Genetic characterization of antiplasmid immunity through a type III-A CRISPR-Cas system. *J. Bacteriol.* 196 310–317. 10.1128/JB.01130-13 24187086 PMC3911255

[B36] Heilbronner S. Hanses F. Monk I. R. Speziale P. Foster T. J. (2013). Sortase a promotes virulence in experimental *staphylococcus lugdunensis* endocarditis. *Microbiology* 159(Pt 10), 2141–2152. 10.1099/mic.0.070292-0 23943787

[B37] Heilbronner S. Holden M. T. G. van Tonder A. Geoghegan J. A. Foster T. J. Parkhill J. (2011). Genome sequence of *Staphylococcus lugdunensis* N920143 allows identification of putative colonization and virulence factors. *FEMS Microbiol. Lett.* 322 60–67. 10.1111/j.1574-6968.2011.02339.x 21682763 PMC3615170

[B38] Hellmark B. Söderquist B. Unemo M. Nilsdotter-Augustinsson Å (2013). Comparison of *Staphylococcus epidermidis* isolated from prosthetic joint infections and commensal isolates in regard to antibiotic susceptibility, *agr* type, biofilm production, and epidemiology. *Int. J. Med. Microbiol. IJMM* 303 32–39. 10.1016/j.ijmm.2012.11.001 23245829

[B39] Higaki S. Kitagawa T. Morohashi M. Yamagishi T. (1999). Distribution and antimicrobial susceptibility of coagulase-negative staphylococci from skin lesions. *J. Int. Med. Res.* 27 191–195. 10.1177/030006059902700406 10599032

[B40] Holden M. T. G. Hsu L.-Y. Kurt K. Weinert L. A. Mather A. E. Harris S. R. (2013). A genomic portrait of the emergence, evolution, and global spread of a methicillin-resistant *Staphylococcus aureus* pandemic. *Genome Res.* 23 653–664. 10.1101/gr.147710.112 23299977 PMC3613582

[B41] Holtfreter S. Grumann D. Schmudde M. Nguyen H. T. T. Eichler P. Strommenger B. (2007). Clonal distribution of superantigen genes in clinical *Staphylococcus aureus* isolates. *J. Clin. Microbiol.* 45 2669–2680. 10.1128/JCM.00204-07 17537946 PMC1951235

[B42] Jarraud S. Lyon G. J. Figueiredo A. M. S. Rard L. G. Vandenesch F. Etienne J. (2000). Exfoliatin-producing strains de?ne a fourth *agr* speci?city group in *Staphylococcus aureus*. *J. Bacteriol* 182:6. 10.1128/jb.182.22.6517-6522.2000 PMC94802 11053400

[B43] Jiang W. Samai P. Marraffini L. A. (2016). Degradation of phage transcripts by CRISPR-associated rnases enables type III CRISPR-Cas immunity. *Cell* 164 710–721. 10.1016/j.cell.2015.12.053 26853474 PMC4752873

[B44] Kavanagh K. Reeves E. P. (2004). Exploiting the potential of insects for *in vivo* pathogenicity testing of microbial pathogens. *FEMS Microbiol. Rev.* 28 101–112. 10.1016/j.femsre.2003.09.002 14975532

[B45] Labrie S. J. Samson J. E. Moineau S. (2010). Bacteriophage resistance mechanisms. *Nat. Rev. Microbiol.* 8 317–327. 10.1038/nrmicro2315 20348932

[B46] Lambe D. W. Jeffery C. Ferguson K. P. Cooper M. D. (1994). Examination of the glycocalyx of four species of *Staphylococcus* by transmission electron microscopy and image analysis. *Microbios* 78 133–143. 8041290

[B47] Lourtet-Hascoët J. Bicart-See A. Félicé M. P. Giordano G. Bonnet E. (2016). Staphylococcus lugdunensis, a serious pathogen in periprosthetic joint infections: comparison to *Staphylococcus aureus* and *Staphylococcus epidermidis*. *Int. J. Infect. Dis.* 51 56–61. 10.1016/j.ijid.2016.08.007 27609028

[B48] Malachowa N. DeLeo F. R. (2010). Mobile genetic elements of *Staphylococcus aureus*. *Cell. Mol. Life Sci.* 67 3057–3071. 10.1007/s00018-010-0389-4 20668911 PMC2929429

[B49] Malachowa N. Whitney A. R. Kobayashi S. D. Sturdevant D. E. Kennedy A. D. Braughton K. R. (2011). Global changes in *Staphylococcus aureus* gene expression in human blood. *PLoS One* 6:e18617. 10.1371/journal.pone.0018617 21525981 PMC3078114

[B50] Marlinghaus L. Becker K. Korte M. Neumann S. Gatermann S. G. Szabados F. (2012). Construction and characterization of three knockout mutants of the *fbl* gene in *Staphylococcus lugdunensis*: characterization of isogenic mutants of *fbl*. *APMIS* 120 108–116. 10.1111/j.1600-0463.2011.02819.x 22229266

[B51] McHardy I. H. Veltman J. Hindler J. Bruxvoort K. Carvalho M. M. Humphries R. M. (2017). Clinical and microbiological aspects of β-lactam resistance in *Staphylococcus lugdunensis*. *J. Clin. Microbiol.* 55 585–595. 10.1128/JCM.02092-16 27927926 PMC5277529

[B52] Michaux C. Sanguinetti M. Reffuveille F. Auffray Y. Posteraro B. Gilmore M. S. (2011). SlyA is a transcriptional regulator involved in the virulence of *Enterococcus faecalis*. *Infect. Immun.* 79 2638–2645. 10.1128/IAI.01132-10 21536798 PMC3191995

[B53] Non L. R. Santos C. A. Q. (2017). The occurrence of infective endocarditis with *Staphylococcus lugdunensis* bacteremia: a retrospective cohort study and systematic review. *J. Infect.* 74 179–186. 10.1016/j.jinf.2016.10.003 27777118

[B54] Novick R. P. Christie G. E. Penadés J. R. (2010). The phage-related chromosomal islands of gram-positive bacteria. *Nat. Rev. Microbiol.* 8 541–551. 10.1038/nrmicro2393 20634809 PMC3522866

[B55] Novick R. P. Ram G. (2017). Staphylococcal pathogenicity islands — movers and shakers in the genomic firmament. *Curr. Opin. Microbiol.* 38 197–204. 10.1016/j.mib.2017.08.001 29100762 PMC5884141

[B56] Novick R. P. Subedi A. (2007). “The SaPIs: mobile pathogenicity islands of *Staphylococcus*,” in *Chemical Immunology and Allergy*, ed. Marone G. (Basel: KARGER), 42–57. 10.1159/000100857 17369699

[B57] O’Riordan K. Lee J. C. (2004). *Staphylococcus aureus* capsular polysaccharides. *Clin. Microbiol. Rev.* 17 218–234. 10.1128/cmr.17.1.218-234.2004 14726462 PMC321462

[B58] Poitevin-Later F. Vandenesch F. Dyke K. Fleurette J. Etienne J. (1992). Cadmium-resistance plasmid in *Staphylococcus lugdunensis*. *FEMS Microbiol. Lett.* 78 59–63. 10.1016/0378-1097(92)90288-y 1468617

[B59] Post V. Harris L. G. Morgenstern M. Mageiros L. Hitchings M. D. Méric G. (2017). Comparative genomics study of *Staphylococcus epidermidis* isolates from orthopedic-device-related infections correlated with patient outcome. *J. Clin. Microbiol.* 55 3089–3103. 10.1128/JCM.00881-17 28794175 PMC5625394

[B60] Poulsen C. Panjikar S. Holton S. J. Wilmanns M. Song Y.-H. (2014). WXG100 protein superfamily consists of three subfamilies and exhibits an a-helical c-terminal conserved residue pattern. *PLoS One* 9:12. 10.1371/journal.pone.0089313 24586681 PMC3935865

[B61] Ramarao N. Nielsen-Leroux C. Lereclus D. (2012). The insect *Galleria mellonella* as a powerful infection model to investigate bacterial pathogenesis. *J. Vis. Exp.* 70:e4392. 10.3791/4392 23271509 PMC3567165

[B62] Roberts R. J. Carneiro M. O. Schatz M. C. (2013). The advantages of SMRT sequencing. *Genome Biol.* 14:405. 10.1186/gb-2013-14-6-405 23822731 PMC3953343

[B63] Roghmann M. Taylor K. L. Gupte A. Zhan M. Johnson J. A. Cross A. (2005). Epidemiology of capsular and surface polysaccharide in *Staphylococcus aureus* infections complicated by bacteraemia. *J. Hosp. Infect.* 59 27–32. 10.1016/j.jhin.2004.07.014 15571850

[B64] Rossi C. C. Souza-Silva T. Araújo-Alves A. V. Giambiagi-deMarval M. (2017). CRISPR-cas systems features and the gene-reservoir role of coagulase-negative staphylococci. *Front. Microbiol.* 8:1545. 10.3389/fmicb.2017.01545 28861060 PMC5559504

[B65] Sabe M. A. Shrestha N. K. Gordon S. Menon V. (2014). *Staphylococcus lugdunensis*?: a rare but destructive cause of coagulase-negative *staphylococcus* infective endocarditis. *Eur. Heart J. Acute Cardiovasc. Care* 3 275–280. 10.1177/2048872614523350 24523355

[B66] Seemann T. (2014). Prokka: rapid prokaryotic genome annotation. *Bioinforma. Oxf. Engl.* 30 2068–2069. 10.1093/bioinformatics/btu153 24642063

[B67] Shah N. B. Osmon D. R. Fadel H. Patel R. Kohner P. C. Steckelberg J. M. (2010). Laboratory and clinical characteristics of *Staphylococcus lugdunensis* prosthetic joint infections. *J. Clin. Microbiol.* 48 1600–1603. 10.1128/JCM.01769-09 20181900 PMC2863876

[B68] Singh V. K. Ring R. P. Aswani V. Stemper M. E. Kislow J. Ye Z. (2017). Phylogenetic distribution and expression of a penicillin-binding protein homologue, ear and its significance in virulence of *Staphylococcus aureus*. *J. Med. Microbiol.* 66 1811–1821. 10.1099/jmm.0.000630 29099691

[B69] Szabados F. Nowotny Y. Marlinghaus L. Korte M. Neumann S. Kaase M. (2011). Occurrence of genes of putative fibrinogen binding proteins and hemolysins, as well as of their phenotypic correlates in isolates of S. lugdunensis of different origins. *BMC Res. Notes* 4:113. 10.1186/1756-0500-4-113 21477287 PMC3089787

[B70] Tan L. Li S. R. Jiang B. Hu X. M. Li S. (2018). Therapeutic targeting of the *Staphylococcus aureus* accessory gene regulator (*agr*) system. *Front. Microbiol.* 9:55. 10.3389/fmicb.2018.00055 29422887 PMC5789755

[B71] Tseng S.-P. Lin Y.-T. Tsai J.-C. Hung W.-C. Chen H.-J. Chen P.-F. (2015). Genotypes and phenotypes of *Staphylococcus lugdunensis* isolates recovered from bacteremia. *J. Microbiol. Immunol. Infect.* 48 397–405. 10.1016/j.jmii.2013.11.006 24388577

[B72] Unnikrishnan M. Constantinidou C. Palmer T. Pallen M. J. (2017). The enigmatic esx proteins: looking beyond mycobacteria. *Trends Microbiol.* 25 192–204. 10.1016/j.tim.2016.11.004 27894646

[B73] van der Mee-Marquet N. Achard A. Mereghetti L. Danton A. Minier M. Quentin R. (2003). *Staphylococcus lugdunensis* infections: high frequency of inguinal area carriage. *J. Clin. Microbiol.* 41 1404–1409. 10.1128/JCM.41.4.1404-1409.2003 12682121 PMC153917

[B74] Wang Y. Hu M. Liu Q. Qin J. Dai Y. He L. (2016). Role of the ESAT-6 secretion system in virulence of the emerging community-associated *Staphylococcus aureus* lineage ST398. *Sci. Rep.* 6:25163. 10.1038/srep25163 27112266 PMC4844983

[B75] Warne B. Harkins C. P. Harris S. R. Vatsiou A. Stanley-Wall N. Parkhill J. (2016). The Ess/Type VII secretion system of *Staphylococcus aureus* shows unexpected genetic diversity. *BMC Genomics* 17:222. 10.1186/s12864-016-2426-7 26969225 PMC4788903

[B76] Whitney J. C. Peterson S. B. Kim J. Pazos M. Verster A. J. Radey M. C. (2017). A broadly distributed toxin family mediates contact-dependent antagonism between gram-positive bacteria. *eLife* 6:e26938. 10.7554/eLife.26938 28696203 PMC5555719

[B77] Wilkinson B. J. Holmes K. M. (1979). *Staphylococcus aureus* cell surface: capsule as a barrier to bacteriophage adsorption. *Infect Immun.* 23 549–552. 154475 10.1128/iai.23.2.549-552.1979 PMC414199

[B78] Yeh C.-F. Chang S.-C. Cheng C.-W. Lin J.-F. Liu T.-P. Lu J.-J. (2016). Clinical features, outcomes, and molecular characteristics of community- and health care-associated *Staphylococcus lugdunensis* Infections. *J. Clin. Microbiol.* 54 2051–2057. 10.1128/JCM.00847-16 27225402 PMC4963507

[B79] Zankari E. Hasman H. Cosentino S. Vestergaard M. Rasmussen S. Lund O. (2012). Identification of acquired antimicrobial resistance genes. *J. Antimicrob. Chemother.* 67 2640–2644. 10.1093/jac/dks261 22782487 PMC3468078

[B80] Zhang D. Iyer L. M. Aravind L. (2011). A novel immunity system for bacterial nucleic acid degrading toxins and its recruitment in various eukaryotic and DNA viral systems. *Nucleic Acids Res.* 39 4532–4552. 10.1093/nar/gkr036 21306995 PMC3113570

[B81] Zipperer A. Konnerth M. C. Laux C. Berscheid A. Janek D. Weidenmaier C. (2016). Human commensals producing a novel antibiotic impair pathogen colonization. *Nature* 535 511–516. 10.1038/nature18634 27466123

