# Supplementary material for: Comparative Genome Analysis of Staphylococcus lugdunensis Shows Clonal Complex-Dependent Diversity of the Putative Virulence Factor, ess/Type VII Locus
Source: Front Microbiol. 2019 Oct 31;10:2479. doi: 10.3389/fmicb.2019.02479 (PMC6834553; doi:10.3389/fmicb.2019.02479)
Supplement: Supplementary file 1 [file Data_Sheet_1.PDF]

Supplementary data

Figure S1. Phylogenetic trees based on (A) the *agr* hypervariable region (*agrB* to *agrD)* and (B) the *cap* hypervariable region (*capH* to *capK*) of the 21 genomes of *S. lugdunensis*.

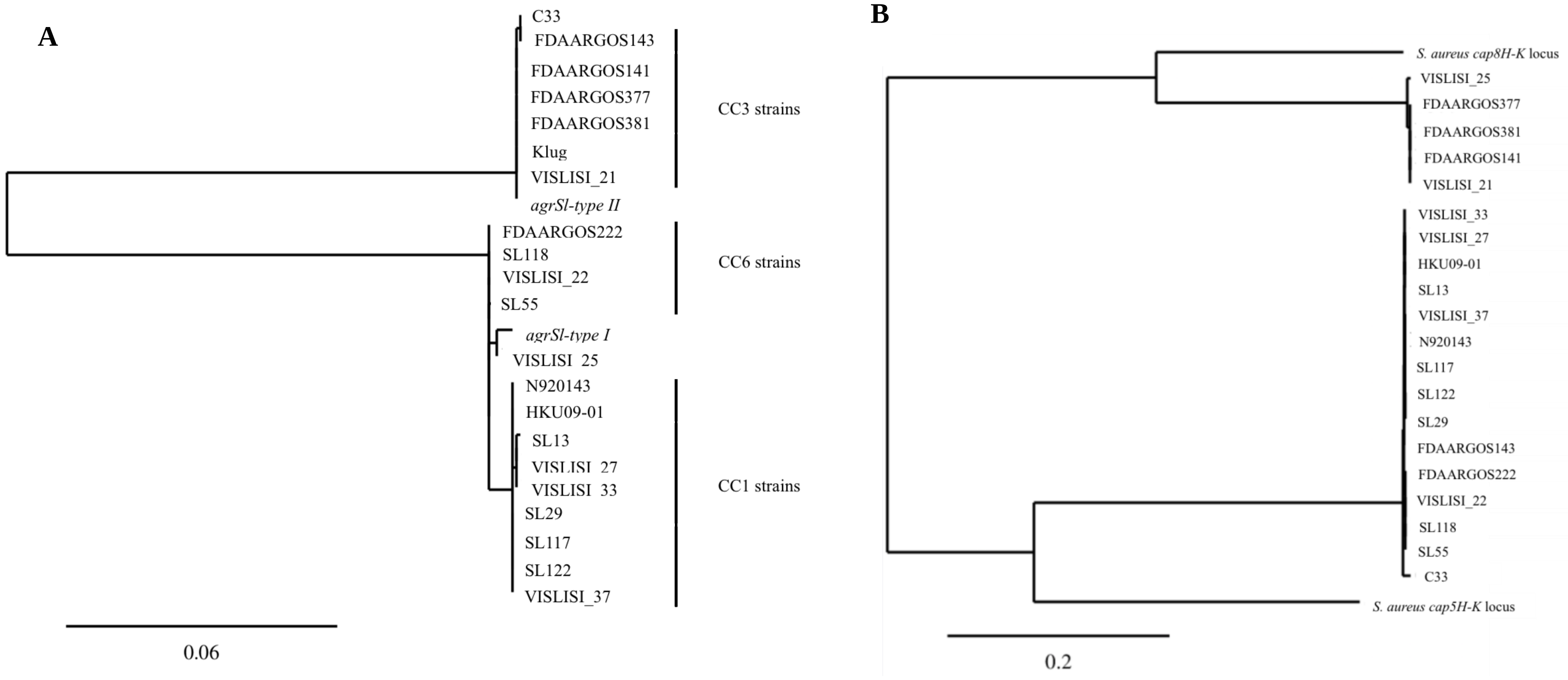

Table S1. Clinical and geographical origins of strains with complete genomes available in NCBI**.**

***S. lugdunensis* strains**	**NCBI accession numbers**	**Isolation source**	**Country**
HKU0901	NC_013893	Skin and soft tissue infection	Hong Kong
N920143	NC_017353	Skin and soft tissue infection	France
FDAARGOS141	NZ_CP014022	Bursitis	USA
FDAARGOS143	NZ_CP014023	Skin and soft tissue infection	USA
FDAARGOS222	NZ_CP020406	Skin and soft tissue infection	Strasbourg
Klug93G4	NZ_CP017069	Carriage	Hong Kong
FDAARGOS377	NZ_CP023539	Abscess	USA
FDAARGOS381	NZ_CP023970	Abscess	USA
VISLISI_21	NZ_CP020762	Bacteraemia	France
VISLISI_22	NZ_CP020764	Infective endocarditis	France
VISLISI_25	NZ_CP020763	Material infection	France
VISLISI_27	NZ_CP020735	Material infection	France
VISLISI_33	NZ_CP020769	Liver abscess	France
VISLISI_37	NZ_CP020761	Infective endocarditis	France
C33	NZ_CP020768	Carriage	France
